# Sensitivity of Bacteria, Protozoa, Viruses, and Other Microorganisms to Ultraviolet Radiation

**DOI:** 10.6028/jres.126.021

**Published:** 2021-08-20

**Authors:** Mahsa Masjoudi, Madjid Mohseni, James R. Bolton

**Affiliations:** 1 Department of Chemical & Biological Engineering University of British Columbia Vancouver, BC, V6T 1Z3 Canada; 2 Department of Civil and Environmental Engineering University of Alberta Edmonton, AB, T6G 2R3 Canada

**Keywords:** algae, bacteria, log reduction, protozoa, spores, ultraviolet, ultraviolet radiation, viruses

## Abstract

Data concerning the sensitivity of various organisms to ultraviolet (UV) radiation exposure are very important in the design of UV disinfection equipment. This review analyzes fluence data from almost 250 studies and organizes the data into a set of recommended fluence values for specific log reductions and an appendix containing all the collected data. This article was sponsored by Dianne L. Poster, Material Measurement Laboratory, and C. Cameron Miller, Physical Measurement Laboratory, National Institute of Standards and Technology (NIST). It is published in collaboration with the International Ultraviolet Association as a complement to the NIST Workshop on Ultraviolet Disinfection Technologies, 14−15 January 2020, Gaithersburg, MD. The views expressed represent those of the authors and not necessarily those of NIST.

## 1 Introduction

This paper represents the third revision of a compilation that goes back to 1999. The original compilation was an internal document of Trojan Technologies [1]. The first revision was published in 2006 [2], and the second revision was published in 2016 [3]. Data from the previous reviews have been included here. In addition, data from the past 5 years (up to March 2021) have been added. Two other reviews of the sensitivity of microorganisms to ultraviolet (UV) radiation have been published elsewhere [4, 5].

Tables A1–A5 (in Appendix A) present, to the best of our knowledge, a summary of all peer-reviewed fluence-response data for UV exposure of various microorganisms that are pathogens, indicators, or organisms encountered in the application, testing of performance, and validation of UV disinfection technologies. The tables reflect the current state of knowledge, but they also include the variation in technique and biological response that currently exists in the absence of standardized protocols (see Refs. 6 and 7). Most of the data are from studies of microorganisms suspended in water; however, there are a few entries for microorganisms on surfaces or in air. Users are encouraged to review the original referenced publication for more details on the experimental protocols before they use the data. The references from which the data were abstracted must be carefully read to understand how the reported fluences were calculated and the assumptions and procedures used in the calculations.

In most cases, the data were generated from low-pressure (LP) monochromatic mercury arc lamp sources for which the lamp fluence rate (irradiance) can be measured empirically and multiplied by exposure time (in seconds) to obtain an incident fluence onto the sample being irradiated. However, earlier data do not always contain the correction factors that are now considered standard practice in order to determine the average fluence delivered to the microorganisms within the irradiated sample [6, 7]. Such uncorrected data are marked and should be considered as upper limits, since the necessary corrections have not been made. Some data are from polychromatic medium-pressure (MP) mercury arc lamps, and in some cases, both lamp types were used. In some cases, filtered polychromatic UV light was used to achieve a narrow band of irradiation around 254 nm. There are also cases where narrow-band light sources, such as UV light-emitting diodes (LEDs) and excilamps (lamps that emit UV radiation when an excited complex, *e.g.*, KrCl* or Xe_2_*, dissociates), have been used. In those cases where the UV exposure was at wavelengths other than 254 nm, the reported fluences have been multiplied by a germicidal factor (GF), defined as the UV sensitivity of a microorganism at wavelength λ normalized to 1.00 at 254 nm. This allows for the fluences to be compared with those using an LP UV lamp at 254 nm. If an action spectrum is available, the GFs were obtained from published action spectra. If no action spectrum is available, the GFs were taken from the relative absorbance of DNA. All GFs were obtained from the review by Bolton [8], except for a few cases where GF values were based on recent data. Note that the GF correction is limited because light sources such as the 222 nm excilamp and UV-LEDs have a significant bandwidth.

*None of the data* incorporated any effect of photorepair processes [9]. Only the response to the inactivating fluence is documented.

It is the intention of the authors and sponsors to maintain this table, with periodic updates. Recommendations for inclusion in the tables, along with the reference sources, should be sent to the authors. The recommended selection criteria for inclusion are the same as those used in the collection of the data in these tables. These criteria are:

1. Data must already be published in a peer-reviewed journal or other peer-reviewed publication media. Some exceptions have been allowed where data are only available in non-peer-reviewed papers.
2. For the publications where an LP or MP UV lamp are used as the UV source, the calculated fluence should usually be determined by using a quasi-collimated beam apparatus; however, for other UV sources, this criterion was not strictly followed, and such cases are noted.
3. Ideally, the fluence rate (irradiance) should have been measured with a recently calibrated radiometer, and when this has not been done, a well-characterized organism should be run as a reference to provide a comparison with the literature values to substantiate that the radiometer is within calibration.
4. The publication from which the data are abstracted should describe the experimental procedures, including collimated beam procedures, fluence calculation procedures along with any assumptions made, organism culturing procedures, and enumeration and preparation for experiments.
5. Ideally, as noted above, the protocol published in Ref. [6] or the recently published International Ultraviolet Association (IUVA) Protocol [7] should be followed. In cases where this protocol has not been followed, notes to that effect have been provided.
6. In some cases, data are provided using a pseudo-monochromatic light source (*e.g.*, UV-LED or excilamp) at wavelengths other than 254 nm. These fluence values have been multiplied by the appropriate GF (see above), so that they can be compared with data obtained using an LP lamp. The GF used is listed in the Notes column of the tables (that is, to recover the value reported in the original reference, divide the value in the table by the GF in the Notes column).
7. Responses should be determined over a range of fluences, that is, a complete fluence-response curve is preferred to a single fluence-response measurement.

These criteria will be applied strictly for future editions of these tables.

For the users of these tables, the following points can be helpful in understanding the information provided:

• In some papers, the authors used different methods for enumeration of their selected microorganism, and based on that, they reported different fluence responses in their work compared with the work of others. Where this has happened for a specific paper, a brief description of the implemented method is provided within the box containing the name of the tested microorganism.
• For the studies with UV sources other than an LP lamp (*e.g.*, filtered MP lamps, UV-LEDs, excilamps), the full width at half maximum (FWHM) of wavelength distribution around the peak wavelength is usually about 10–12 nm, except for the tunable laser, where the bandwidth is less than 1 nm.
• Where the authors have reported kinetic models based on their experimental data, these models were used in fluence calculations for these tables. Where model fits were not provided, the fluence reported for each specific log reduction number was extracted by graphic linearization (World-Wide-Web plot digitizer software) between two adjacent experimental data points in the fluence range.
• In some cases, fluence-response curves have been determined at several wavelengths, so that an action spectrum can be determined. These cases are noted as “action spectrum”; however, only data for wavelengths near 254 nm are included in the tables. Data for other wavelengths can be obtained from the cited reference.
• The reader should be aware that for a given microorganism, there is a data spread even after the selection criteria have been applied. Some studies have applied a Bayesian statistical analysis (*e.g.*, see Refs. [10, 11]) to obtain an average fluence-response curve and 95 percentile limits. Some of the factors that could affect the reported data are: the medium (*e.g.*, drinking water or wastewater), differences in the nutritional state of the cells being assayed, the presence of particles because of a failure to fully disperse cells following preconcentration for the collimated beam assay, *etc*.
• For a given microorganism, the fluence-response curve can depend markedly on the strain examined. This is why studies of a given strain have been grouped together.
• Note that the data in the tables below originate from highly controlled protocols usually using defined media and culture methods, irradiation methods, *etc*. These data are useful when validating UV technologies and envisioning regulations; however, as water quality, growth-phase state, particle content, and a number of other factors can impact microbe responses to disinfection in real environmental samples or processed water, such real waters should be used for site-specific assessments of UV disinfection, and design specification should benefit from the results of assays using these site-specific waters.
• In some cases, the quality of the data was questionable and did not meet some of the selection criteria listed above. In these cases, the data entries are in italics.
• In some cases, errors are given; these are usually at the 95% confidence level

These tables can be used as a helpful document for understanding the fluence responses of different microorganisms at various wavelengths, with different UV sources; however, if more details are important for the users of these data, they must read the reference provided for each study.

Throughout this review, fluence rate and irradiance (units mW cm^–2^) are used interchangeably, since they are virtually identical in a quasi-collimated beam apparatus. The term fluence (in units of mJ cm^–2^) is used, which is the proper term [see Ref. [12] for a recommended set of terms and definitions] rather than “UV dose,” which was used in earlier revisions of this document; however, it should be noted that the term UV dose is still widely used. Finally, it is noted that in Europe and other parts of the world, the units W m^–2^ for irradiance or fluence rate and J m^–2^ for fluence (UV dose) are more commonly used; the conversions are 1 mW cm^–2^ = 10 W m^–2^ and 1 mJ cm^–2^ = 10 J m^–2^.

The data in the tables are for specific log reductions, where log reduction = 1, 2, 3, 4, and 5 for mean 90%, 99%, 99.9%. 99.99%, and 99.999% reduction, respectively. Log reduction is defined as log_10_ (*N*_0_/*N*), where *N*_0_ is the initial viable microorganism count, and *N* is the final value after UV exposure.

## 2 Recommended Tables

In this review, for the first time, we have provided a table of recommended values, with the complete data set in Appendix A. The criteria for selecting recommended values were:

• Among various studies of a given microorganism/strain, a certain publication exhibited a very careful analysis that was deemed reliable.
• In some cases, data are available from a very large data set of fluence-response curves. In these cases, the entry in the recommendation table is highlighted in boldface type. These cases should be considered as *standard* values for that microorganism/strain.

Five tables of recommended values (Tables 1–5) cover spores, bacteria, protozoa, viruses, and algae and other large microorganisms.

**Table 1:**
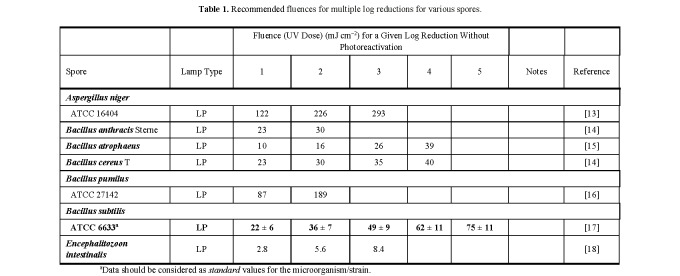
Recommended fluences for multiple log reductions for various spores.

a Data should be considered as *standard* values for the microorganism/strain.

**Table 2:**
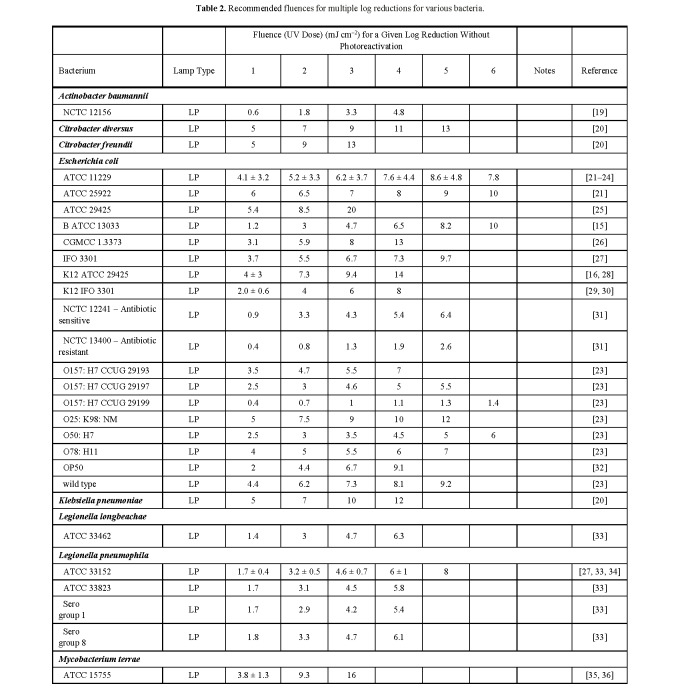

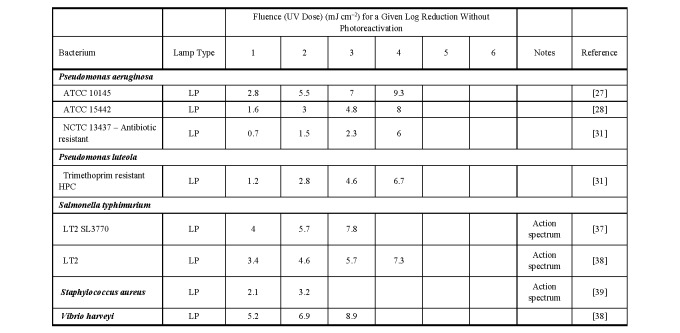
Recommended fluences for multiple log reductions for various bacteria.

**Table 3:**
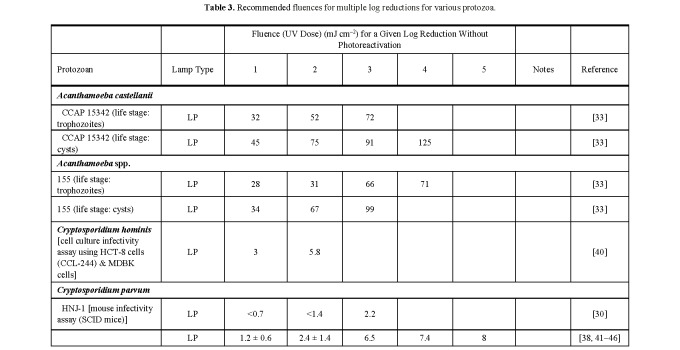

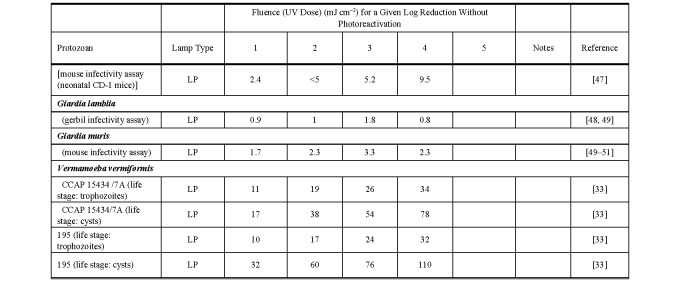
Recommended fluences for multiple log reductions for various protozoa.

**Table 4:**
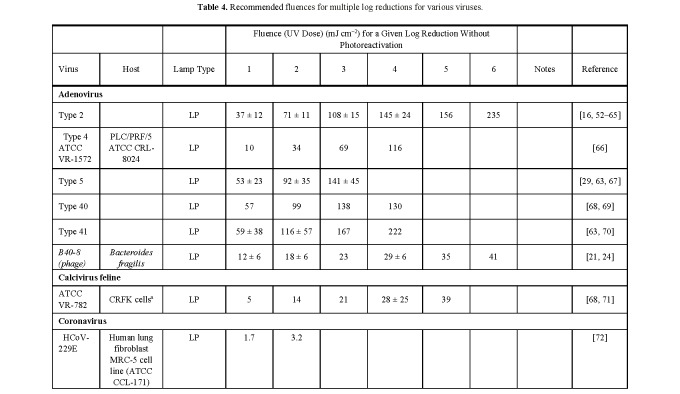

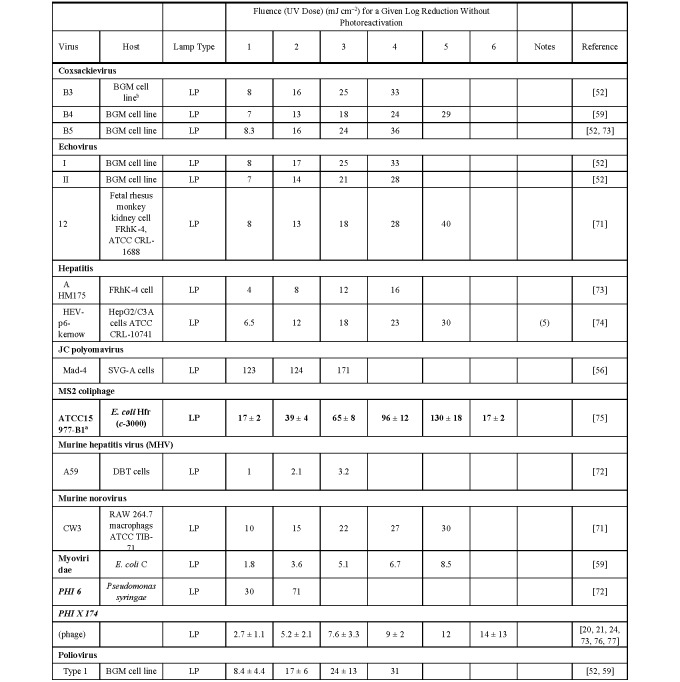

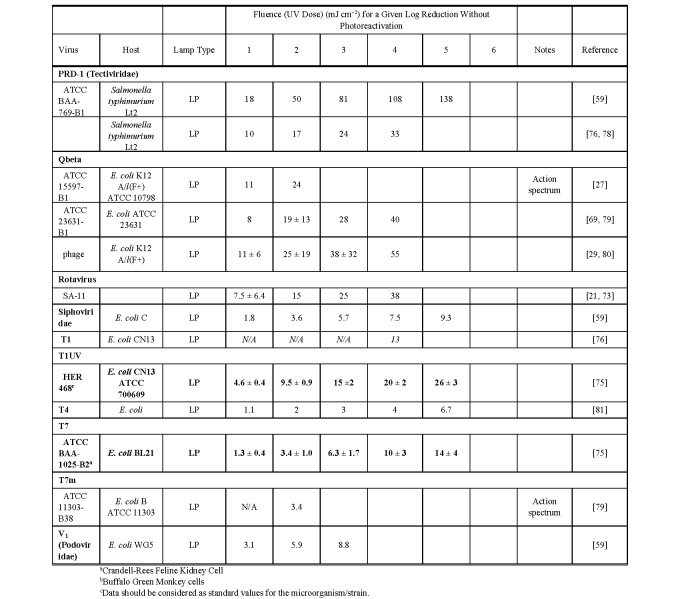
Recommended fluences for multiple log reductions for various viruses.

a Crandell-Rees Feline Kidney Cell

b Buffalo Green Monkey cells

c Data should be considered as standard values for the microorganism/strain.

**Table 5:**
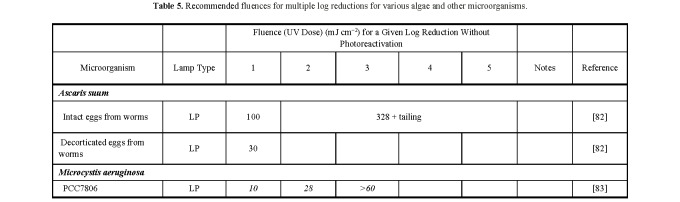
Recommended fluences for multiple log reductions for various algae and other microorganisms.

## Figures and Tables

**Table A1:**
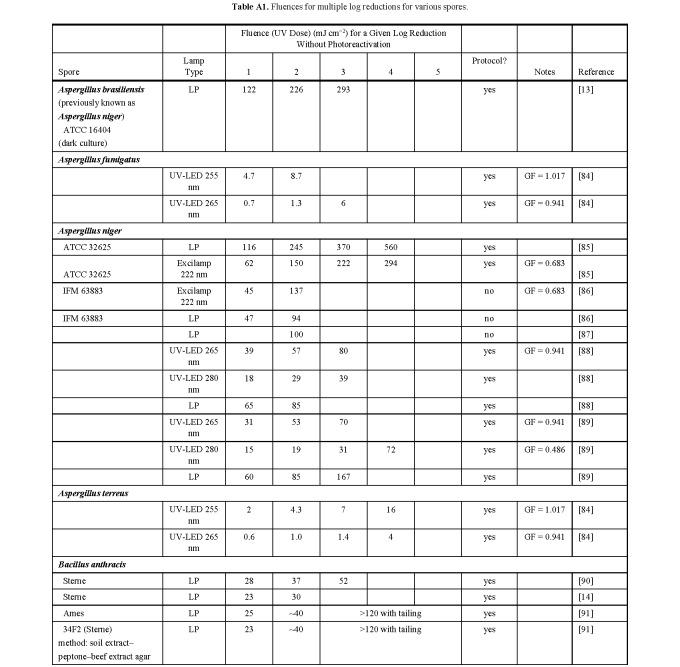

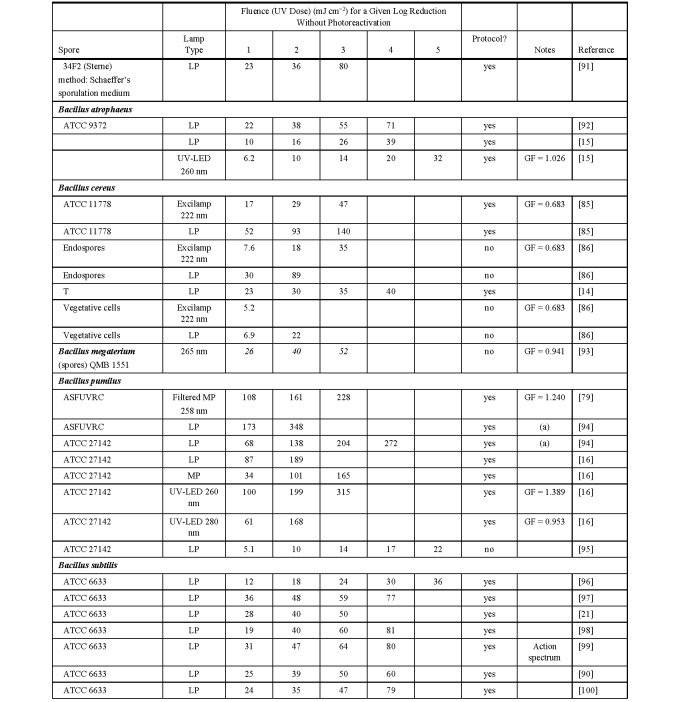

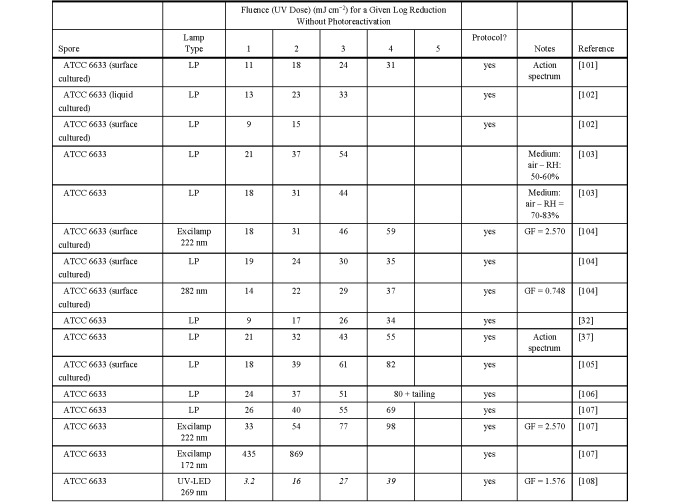

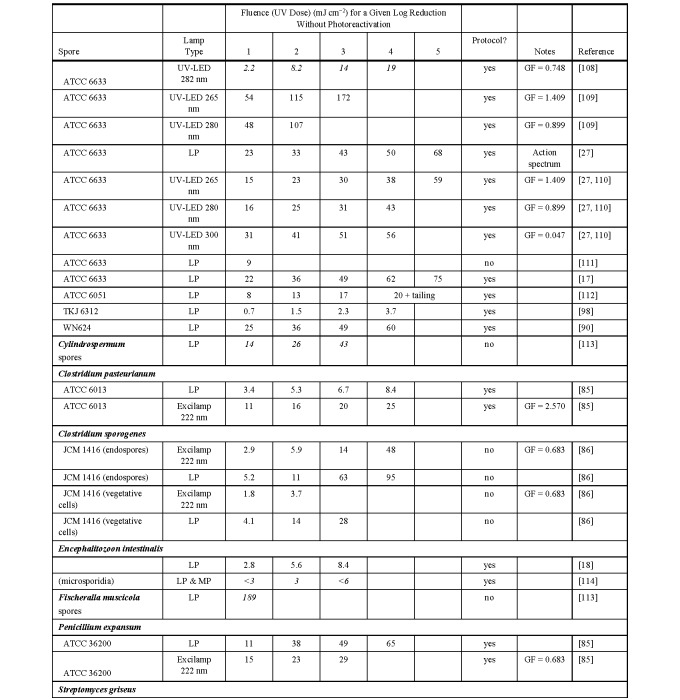

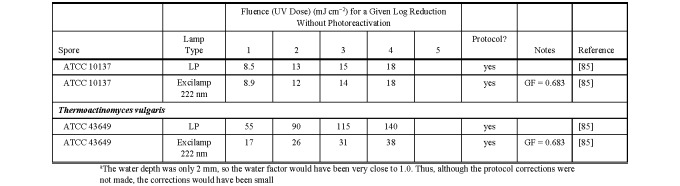
Fluences for multiple log reductions for various spores.

a The water depth was only 2 mm, so the water factor would have been very close to 1.0. Thus, although the protocol corrections were not made, the corrections would have been small

**Table A2:**
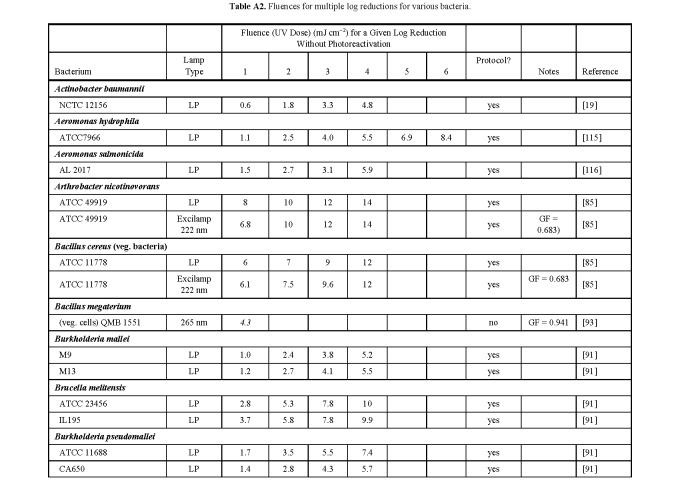

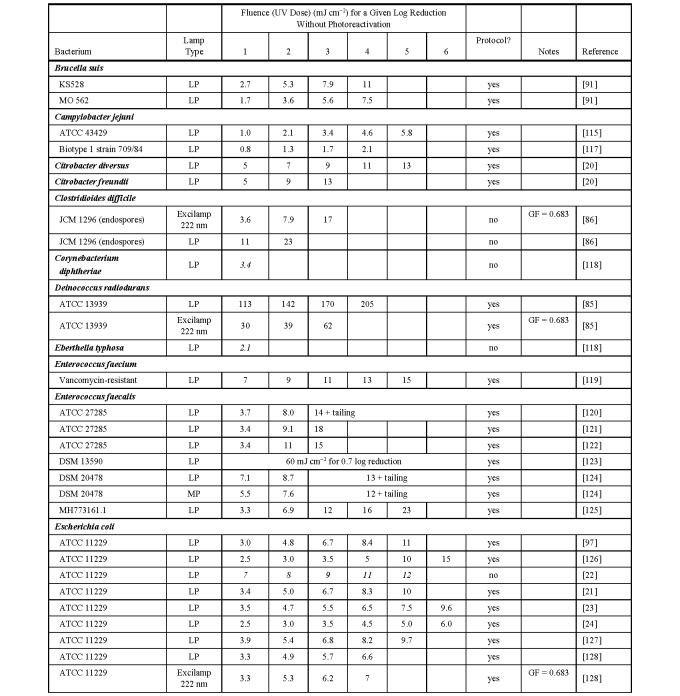

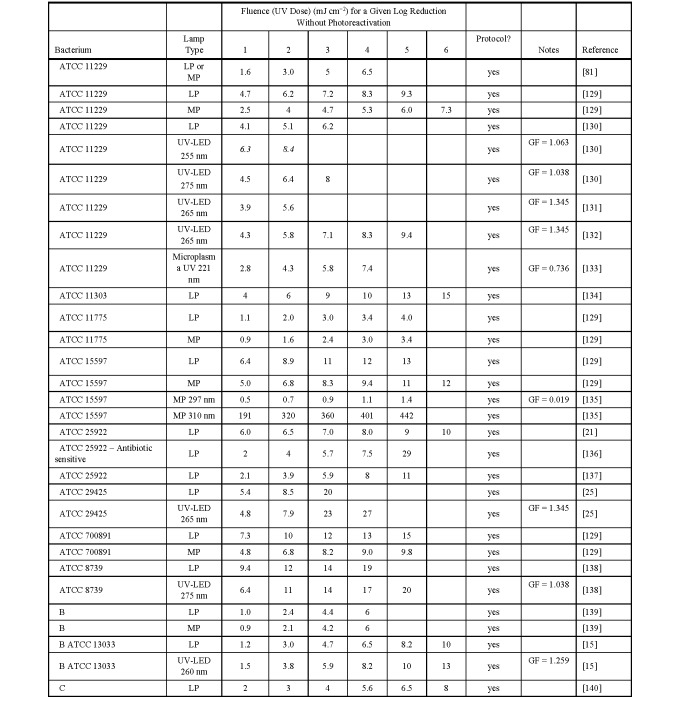

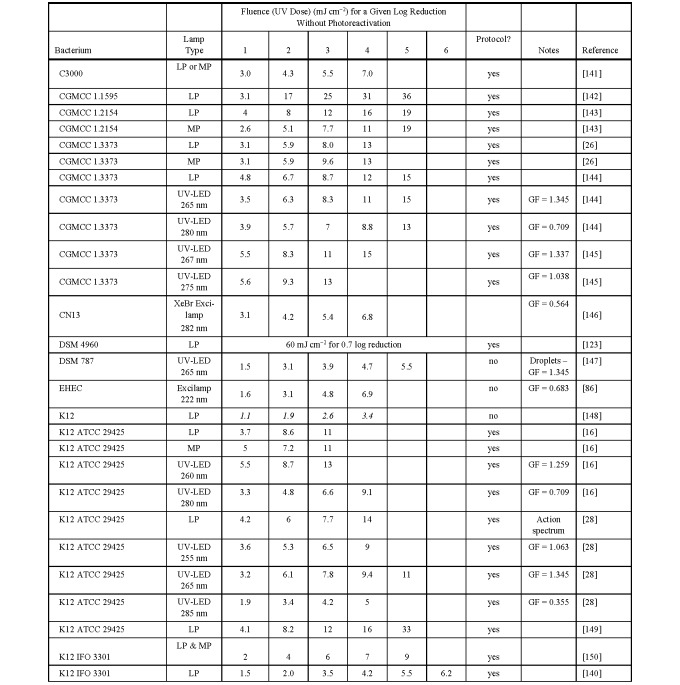

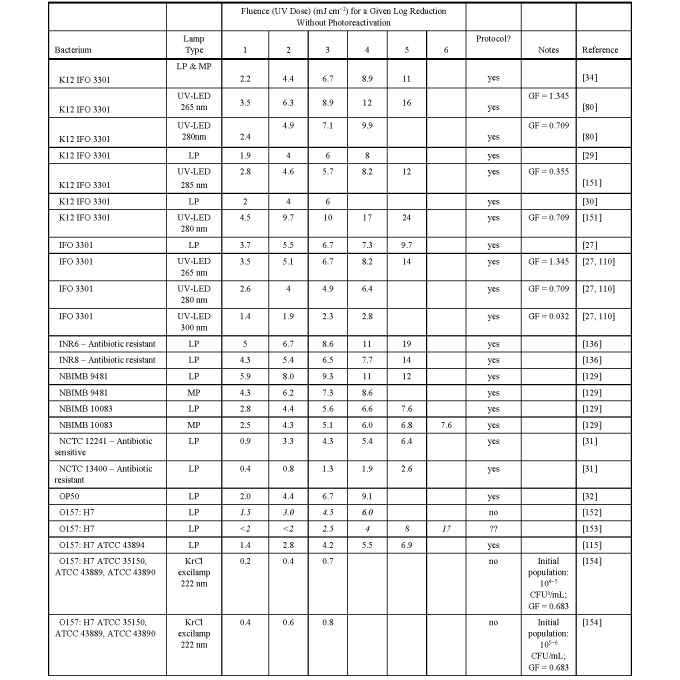

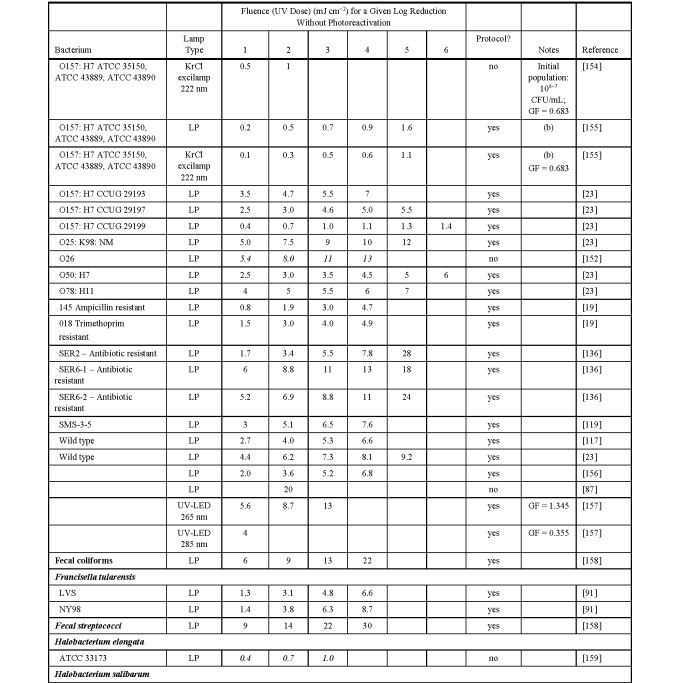

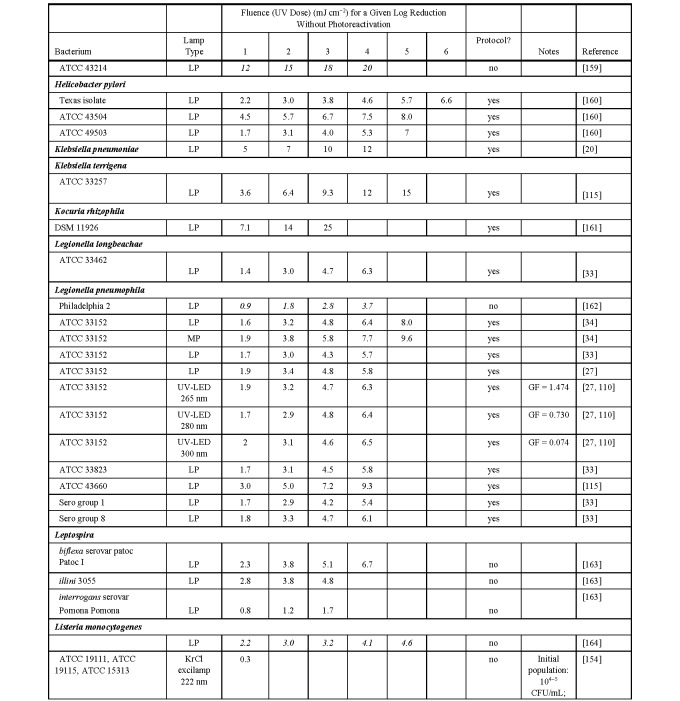

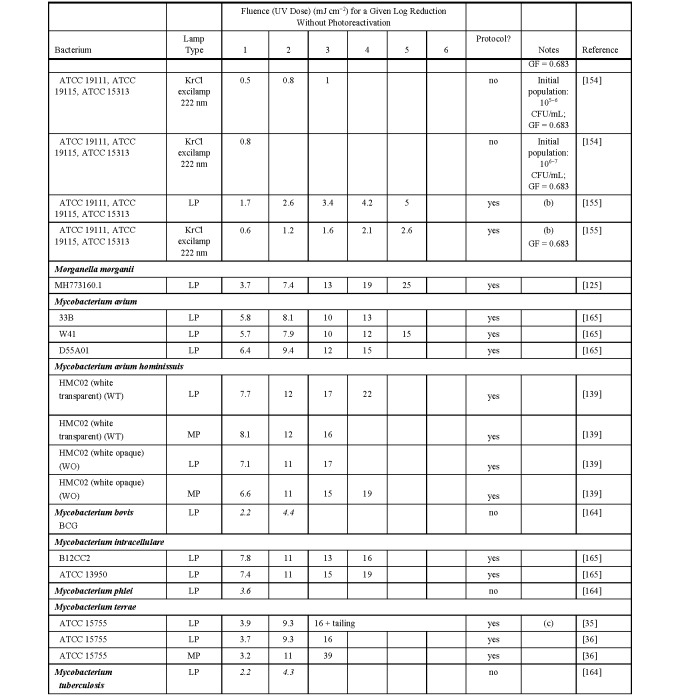

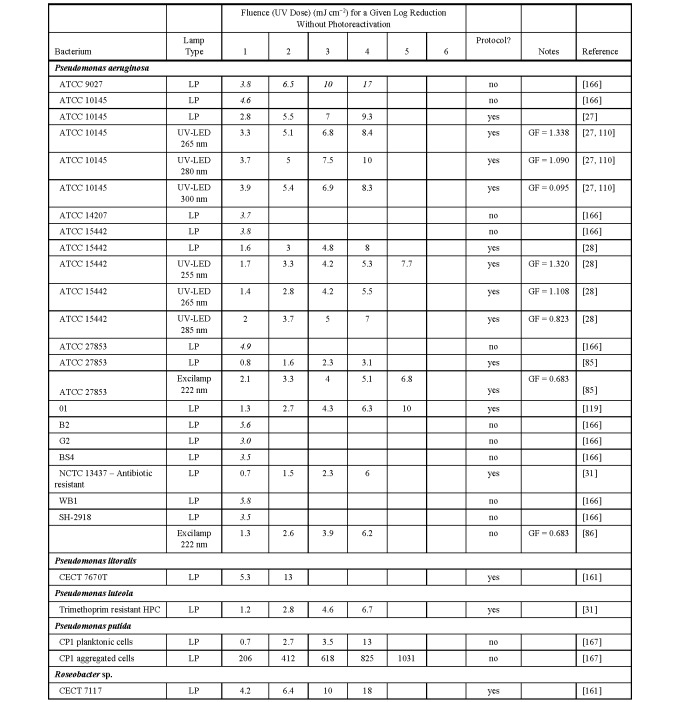

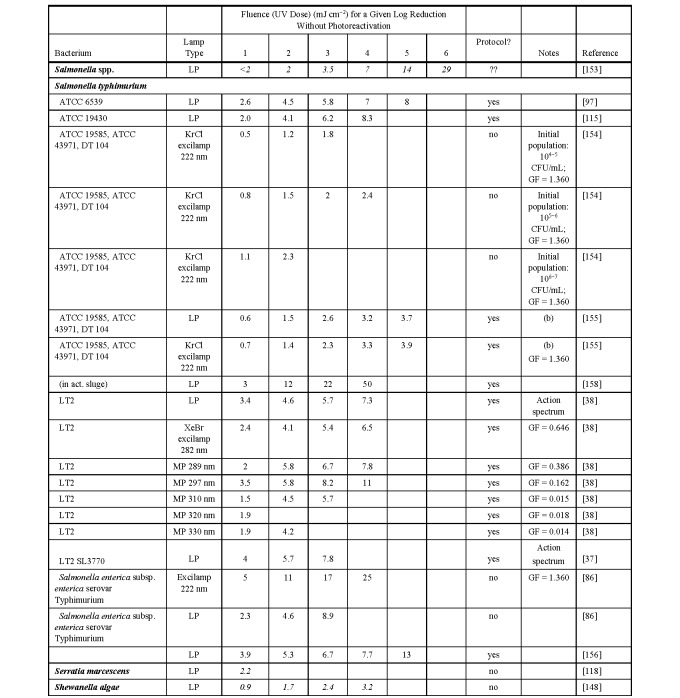

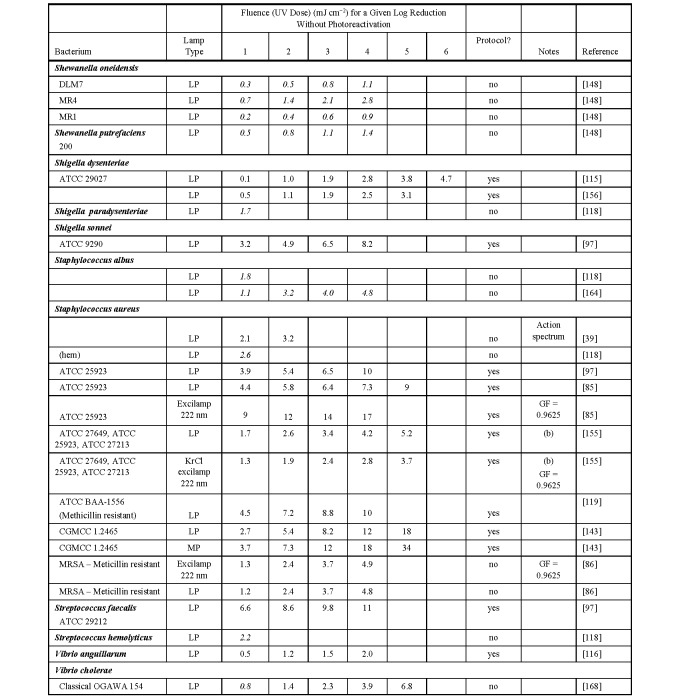

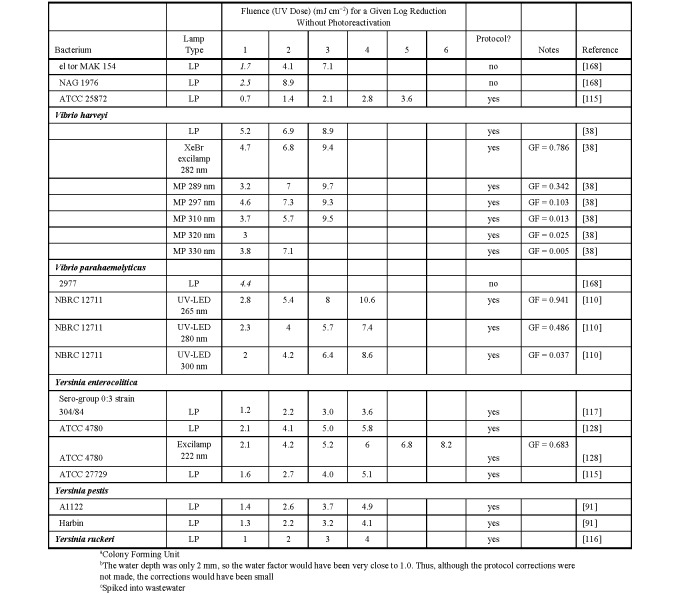
Fluences for multiple log reductions for various bacteria.

a Colony Forming Unit

b The water depth was only 2 mm, so the water factor would have been very close to 1.0. Thus, although the protocol corrections were not made, the corrections would have been small

c Spiked into wastewater

**Table A3:**
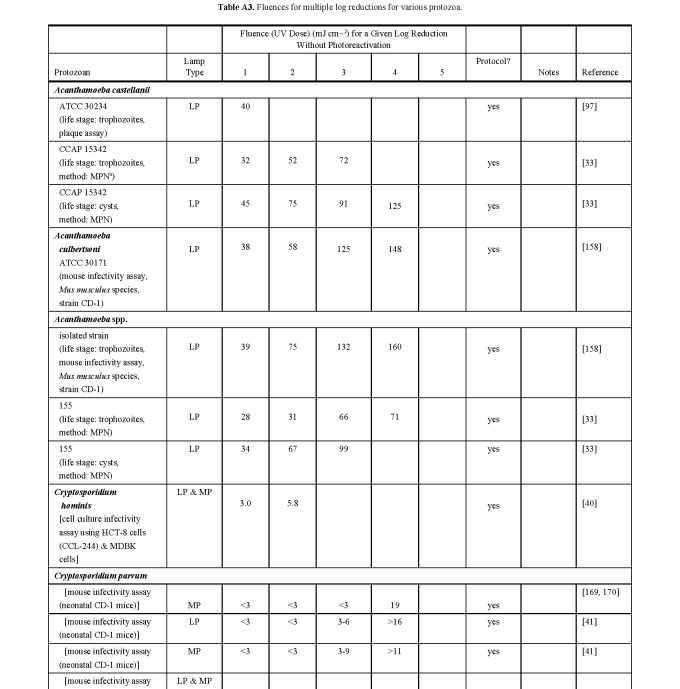

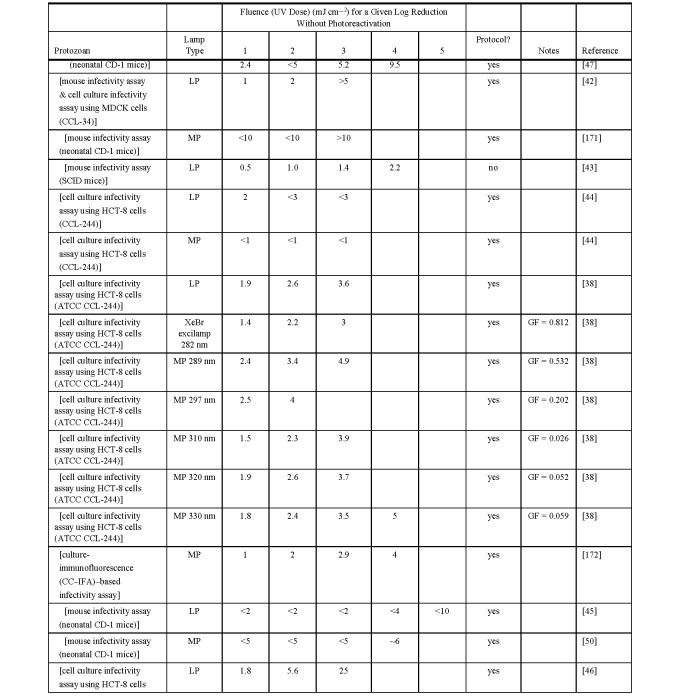

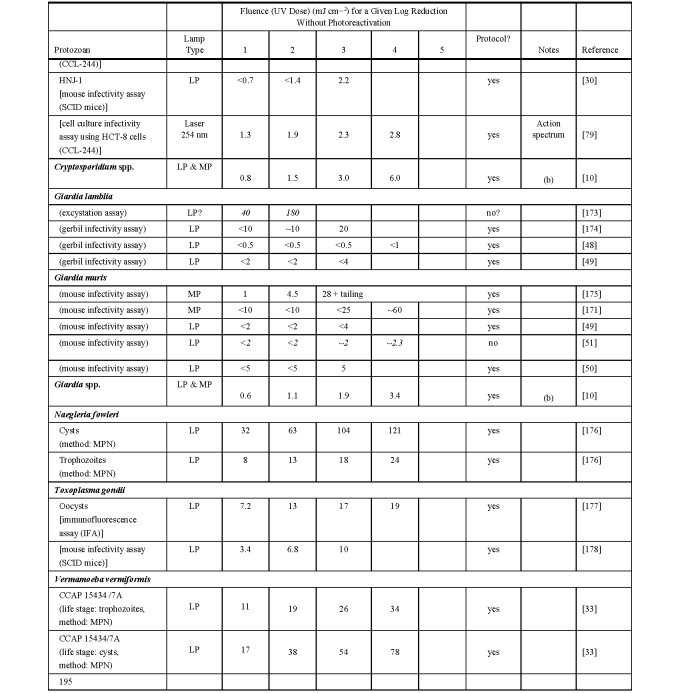

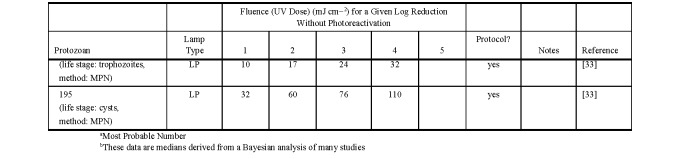
Fluences for multiple log reductions for various protozoa.

a Most Probable Number

b These data are medians derived from a Bayesian analysis of many studies

**Table A4:**
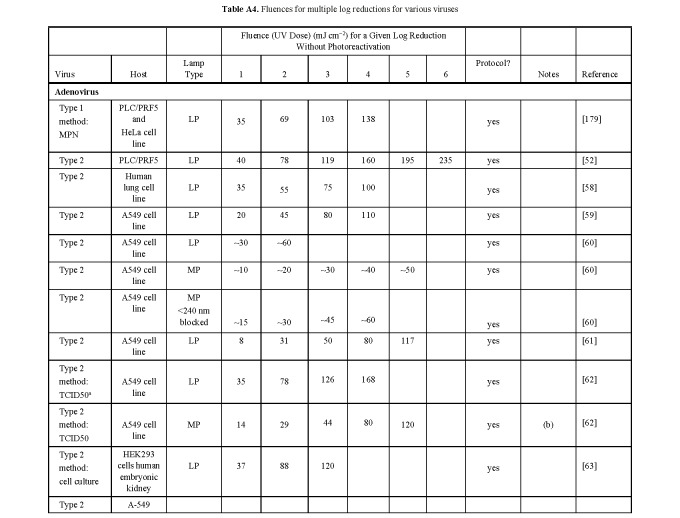

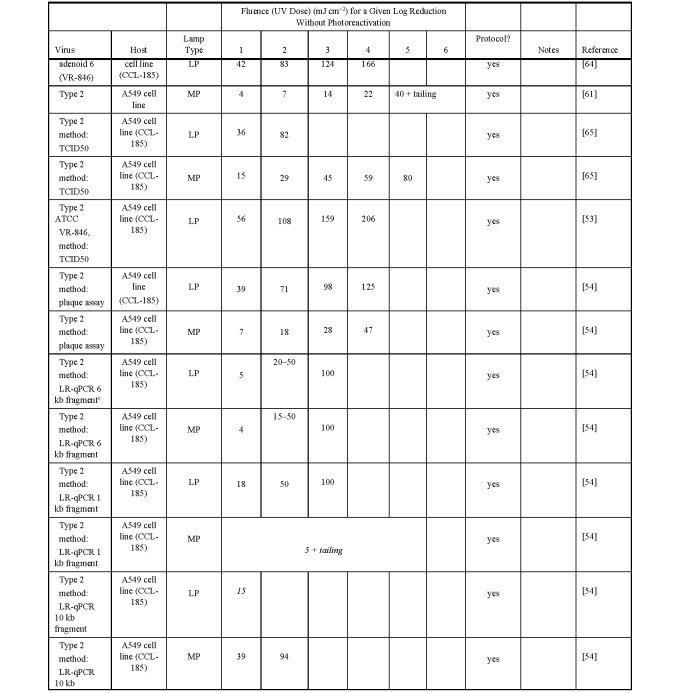

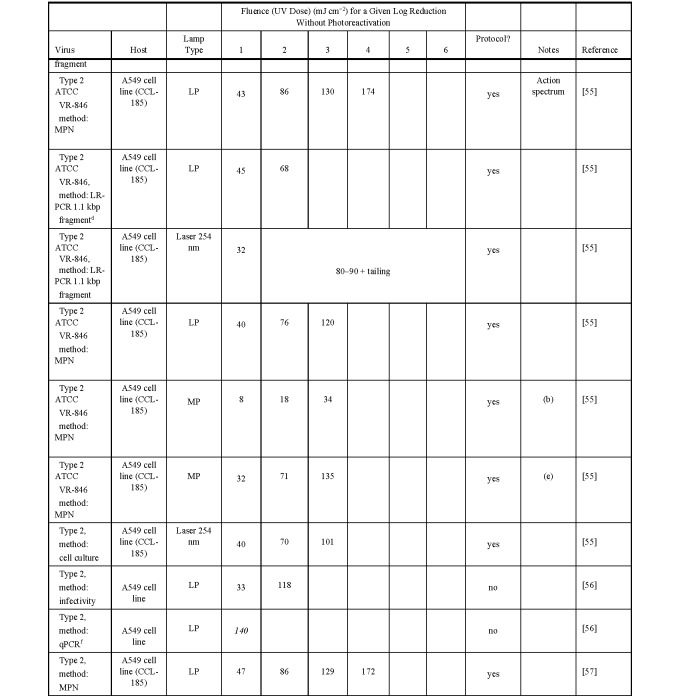

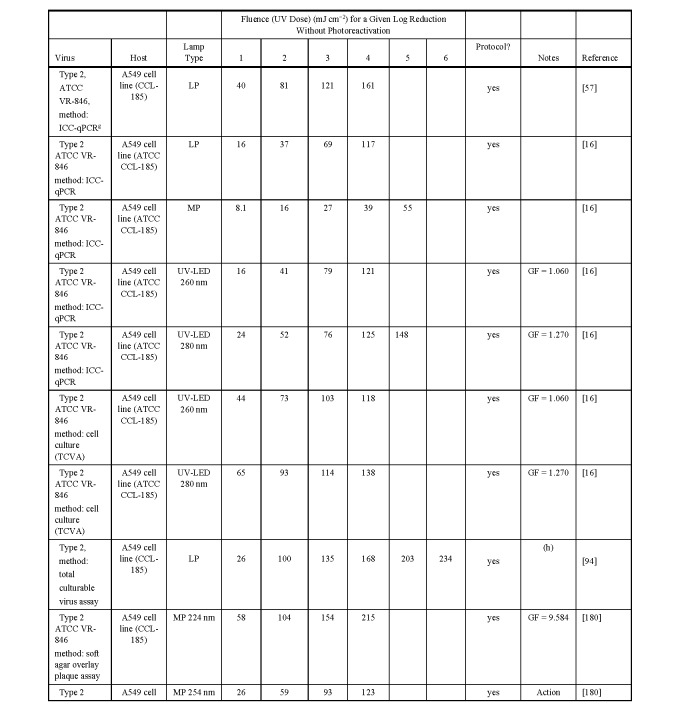

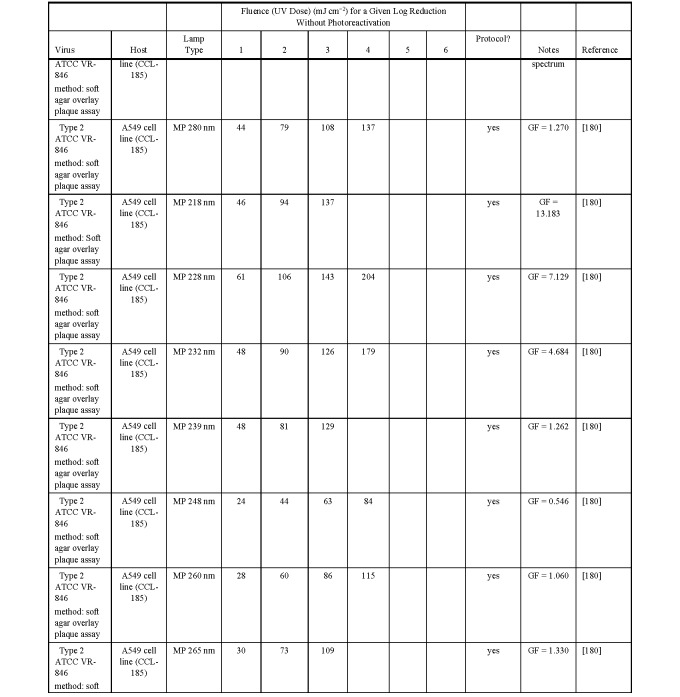

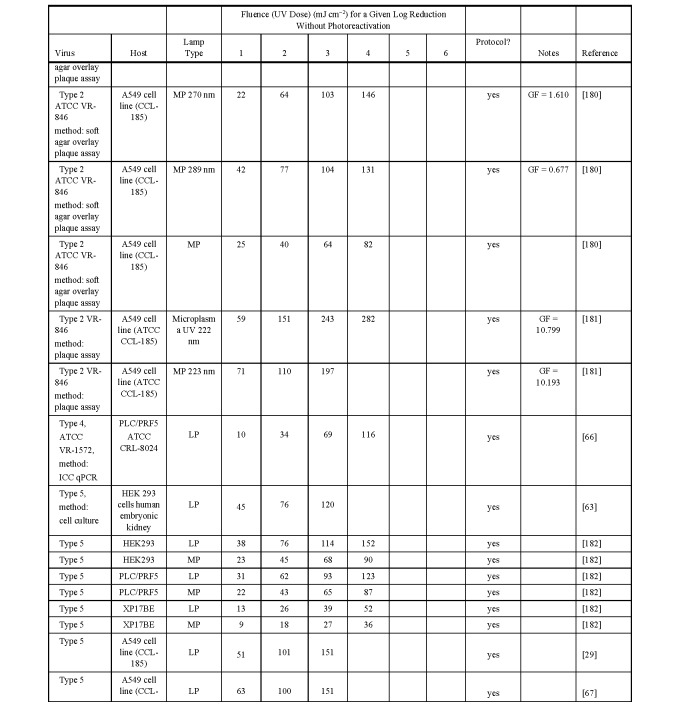

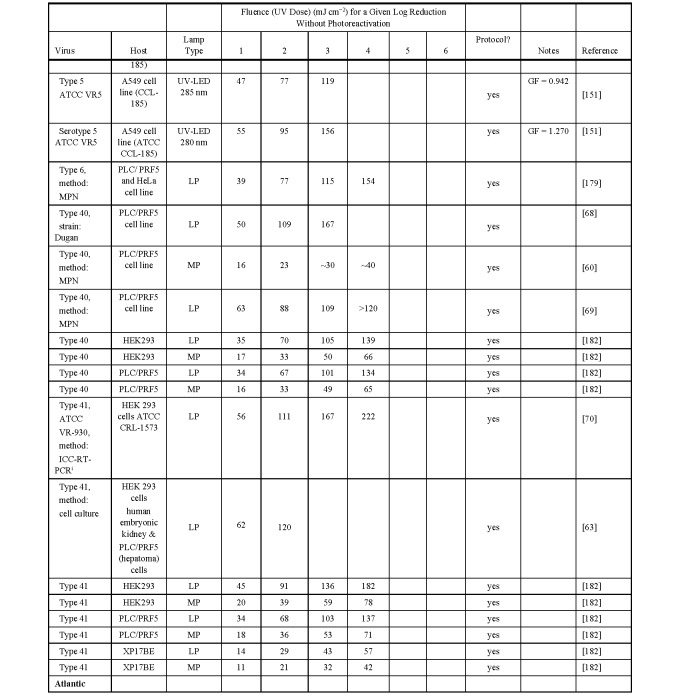

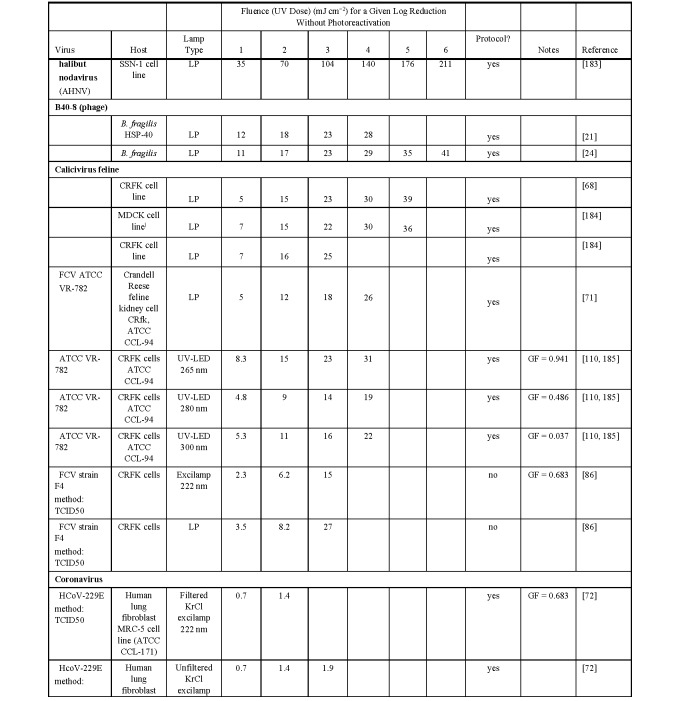

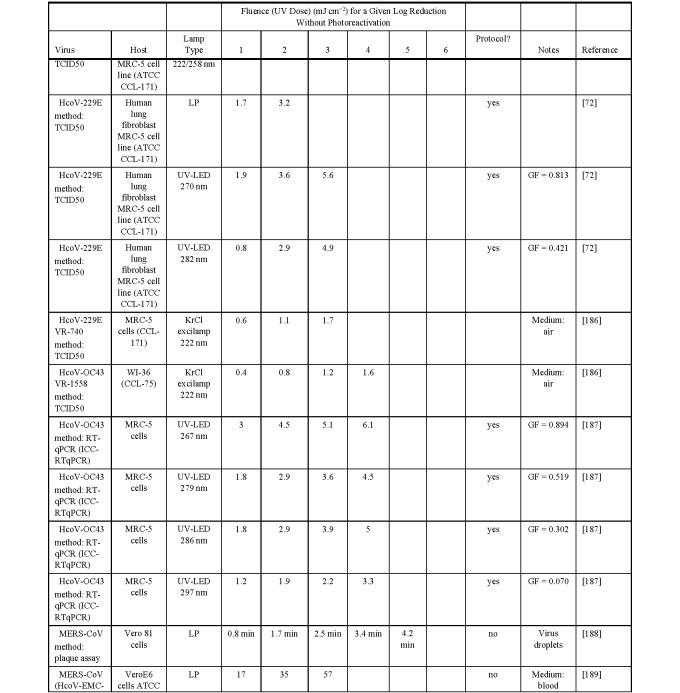

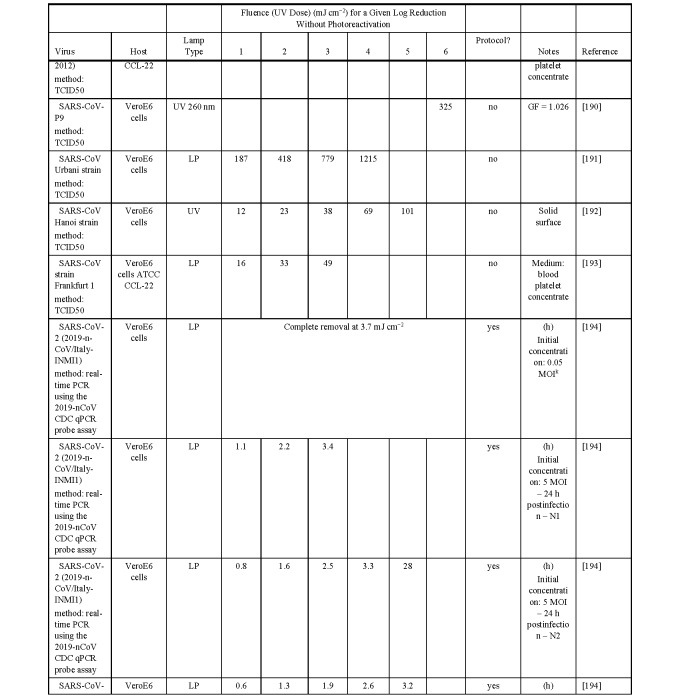

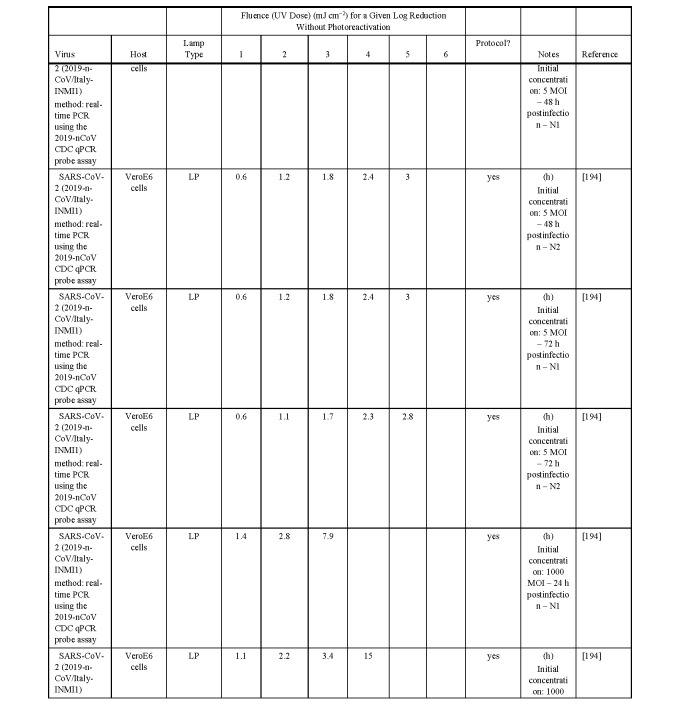

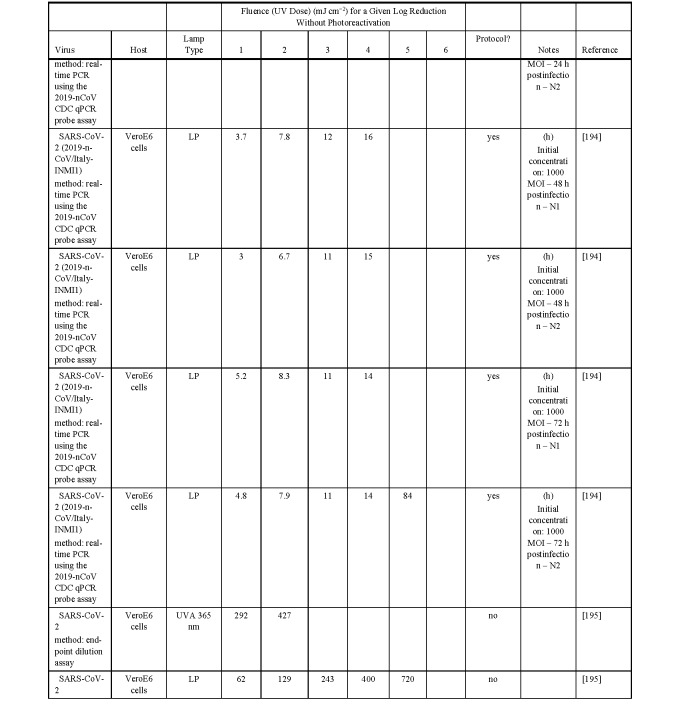

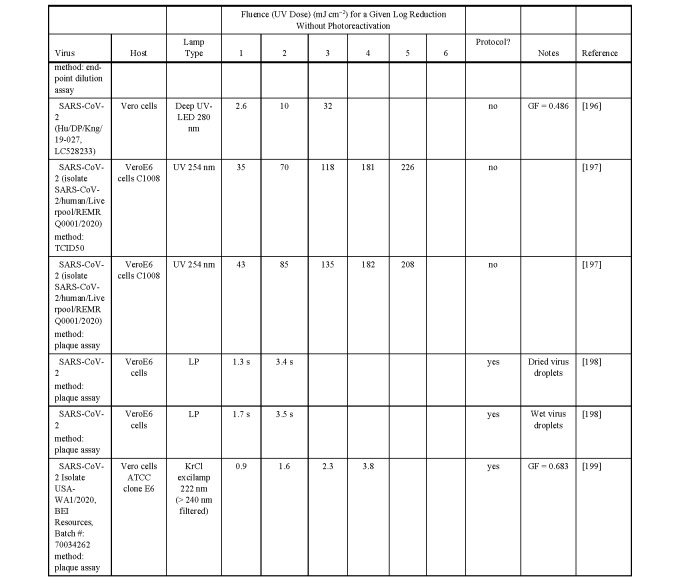

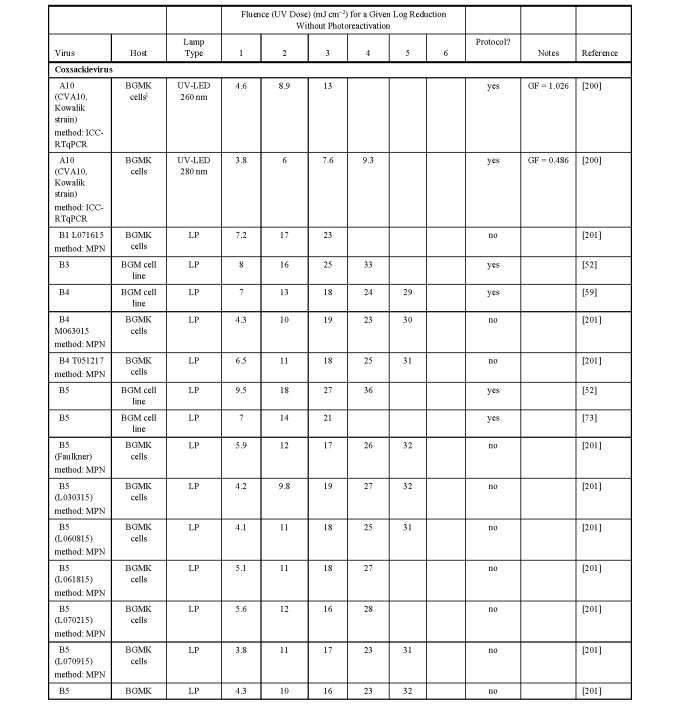

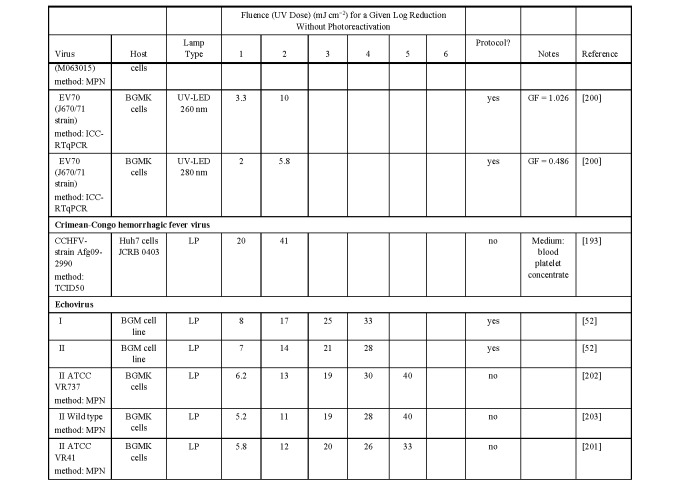

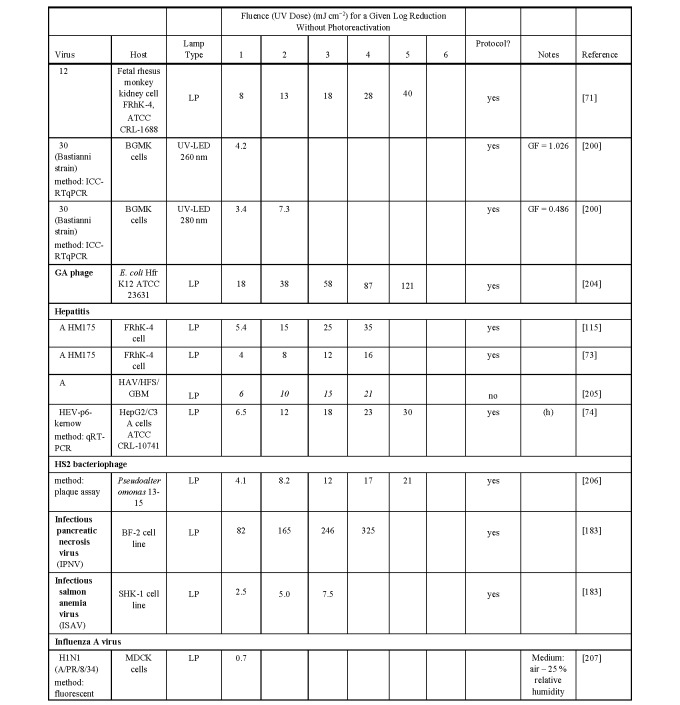

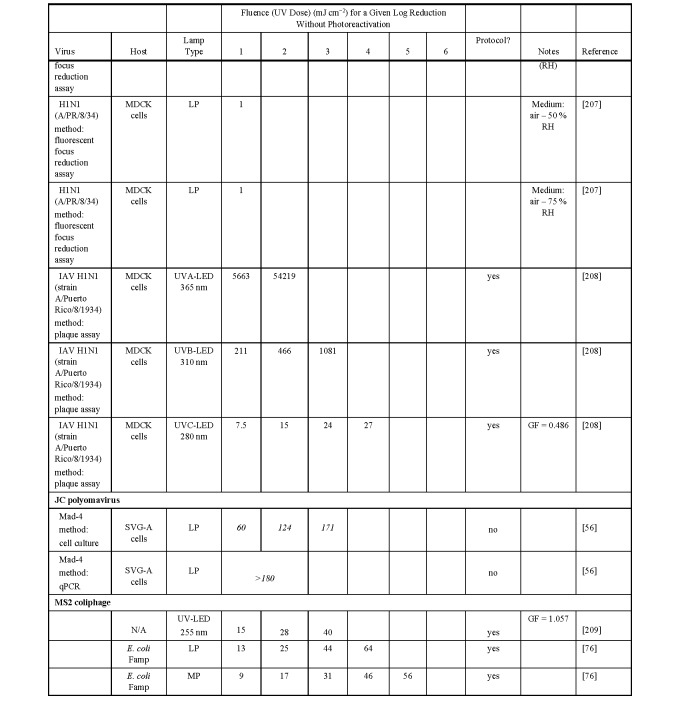

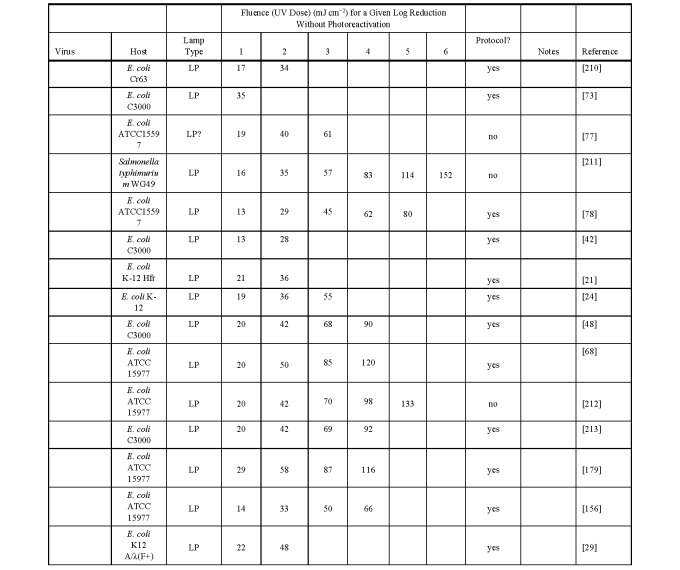

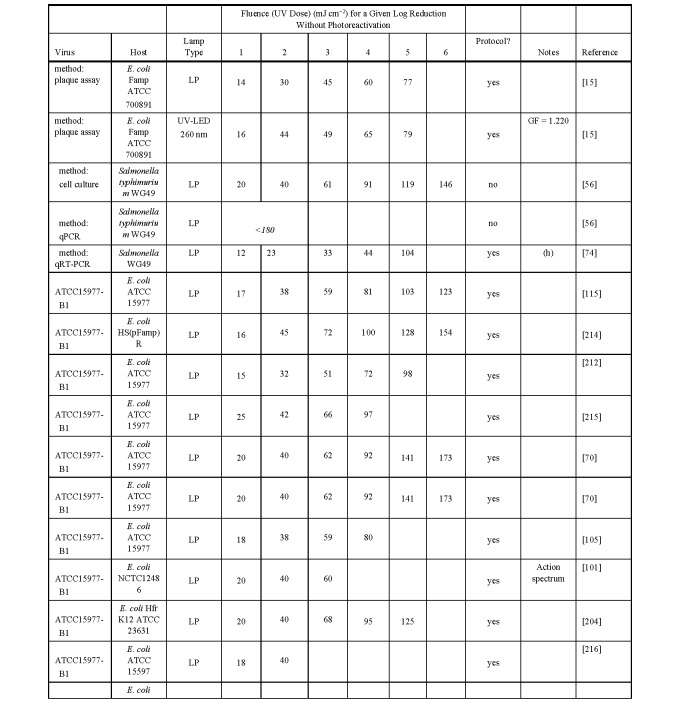

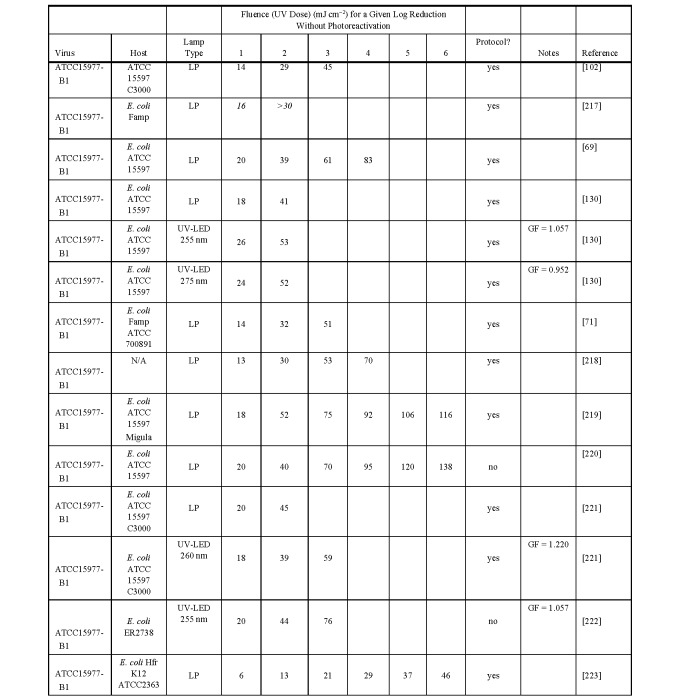

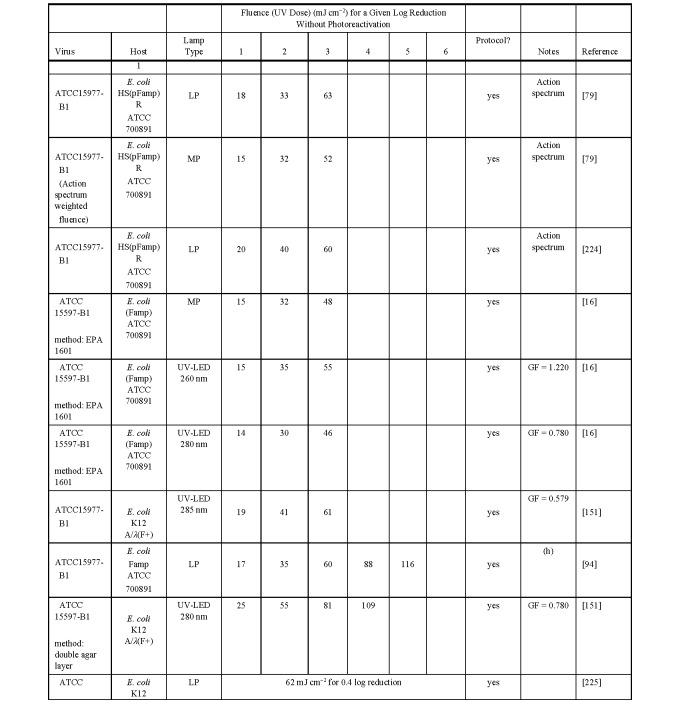

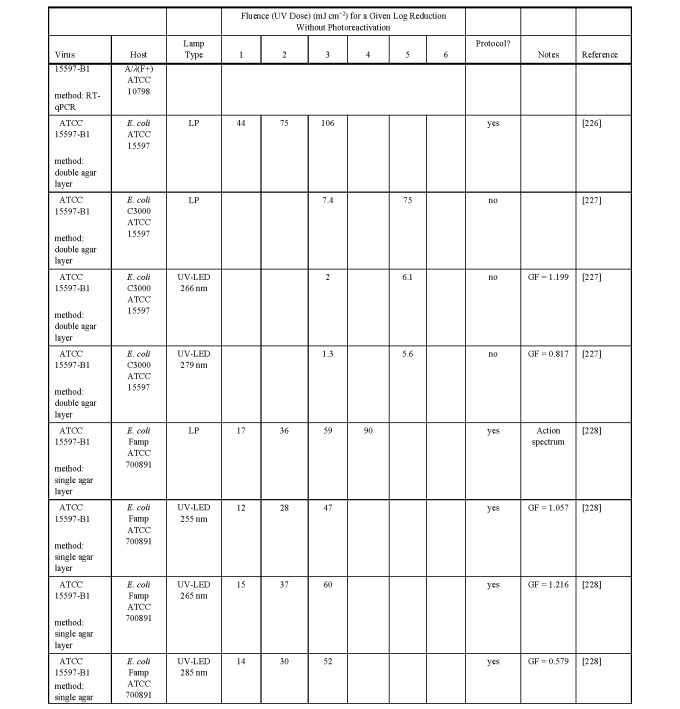

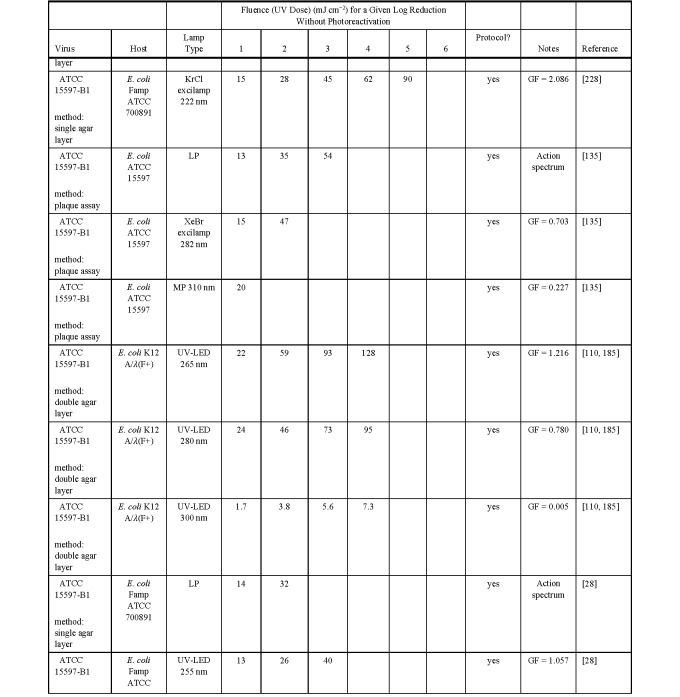

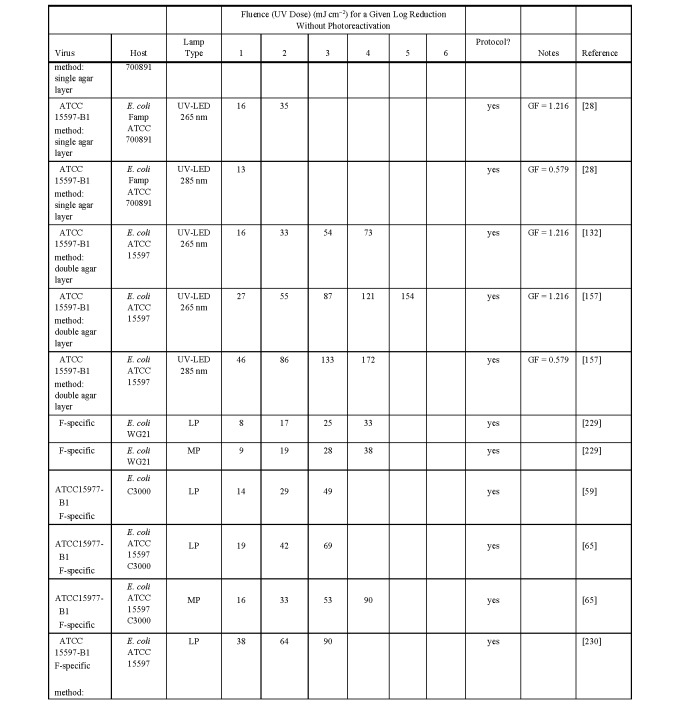

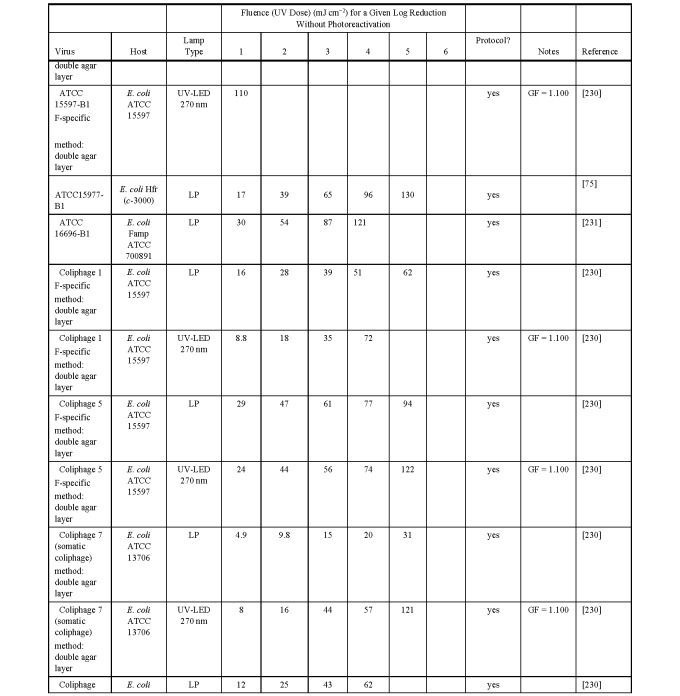

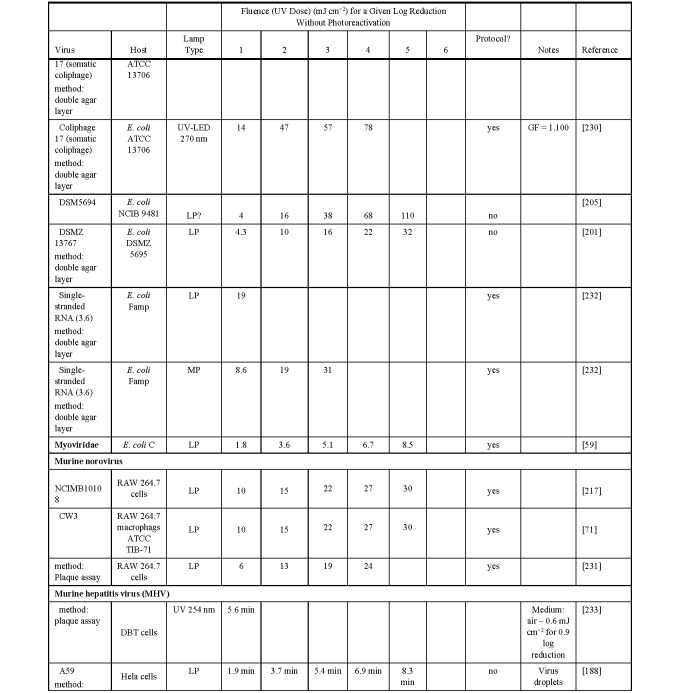

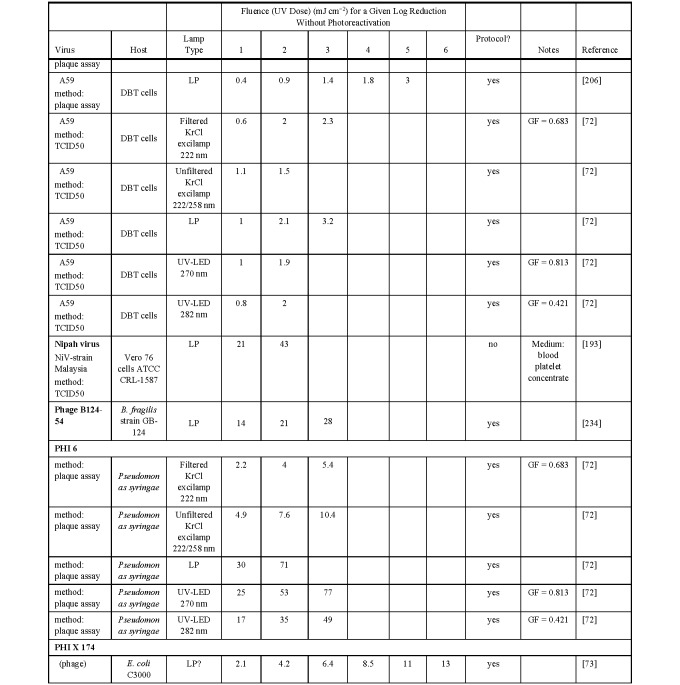

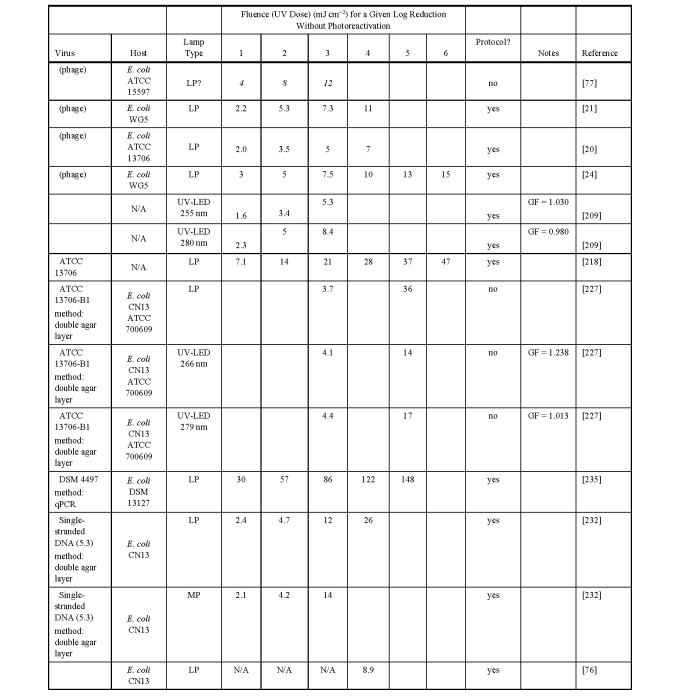

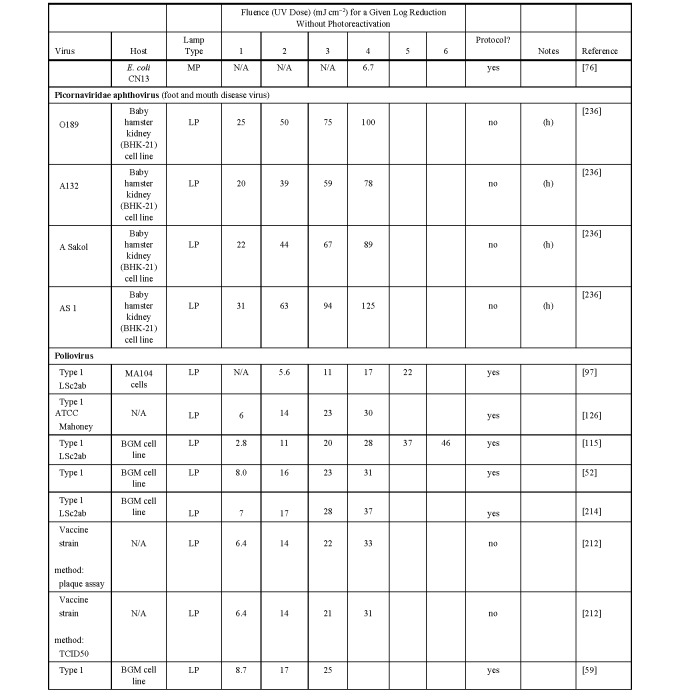

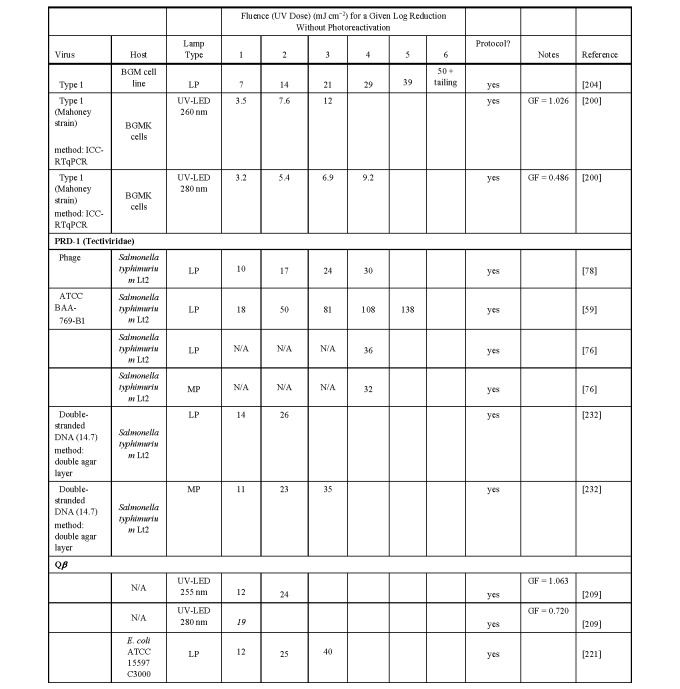

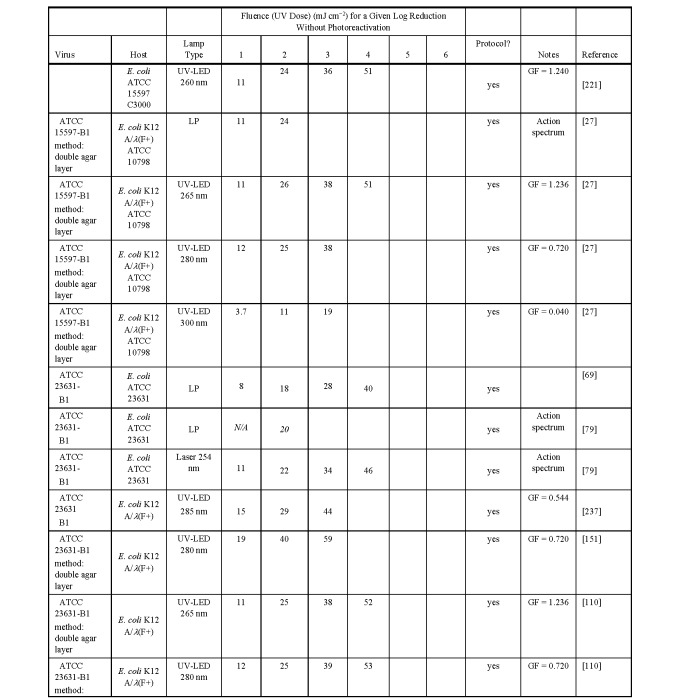

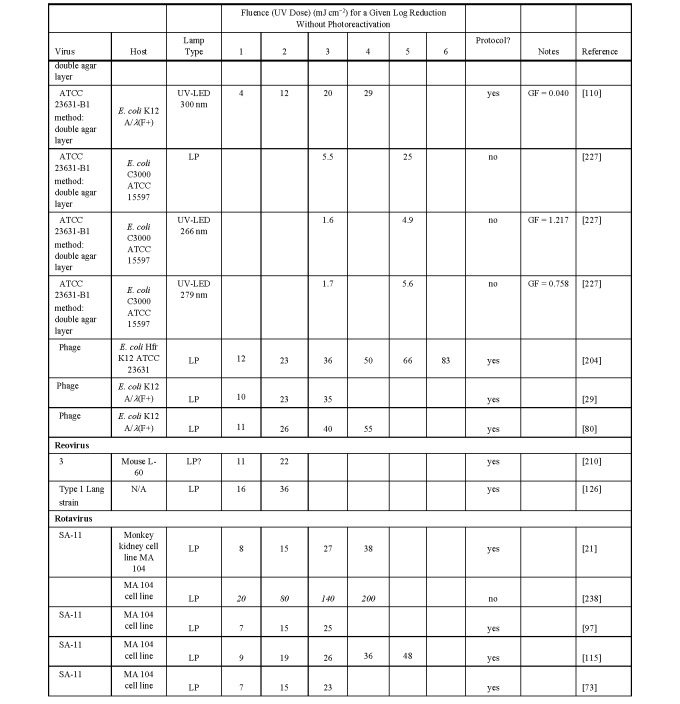

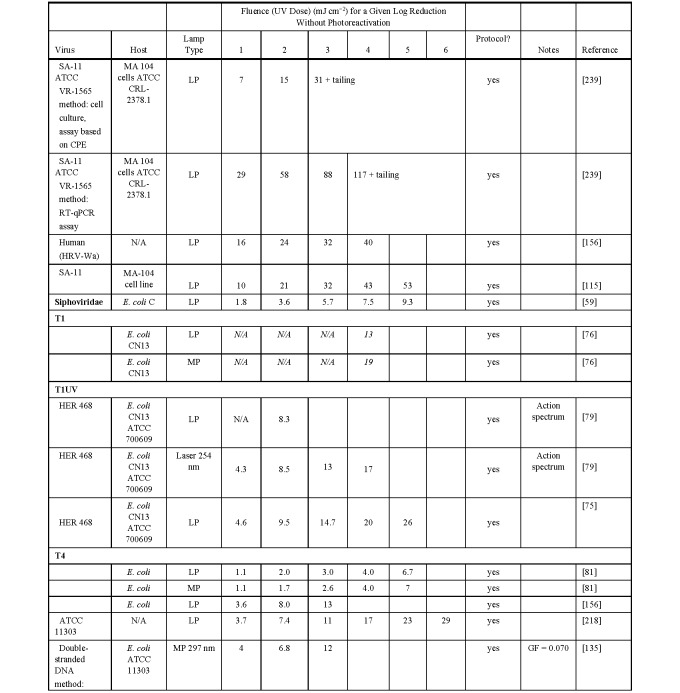

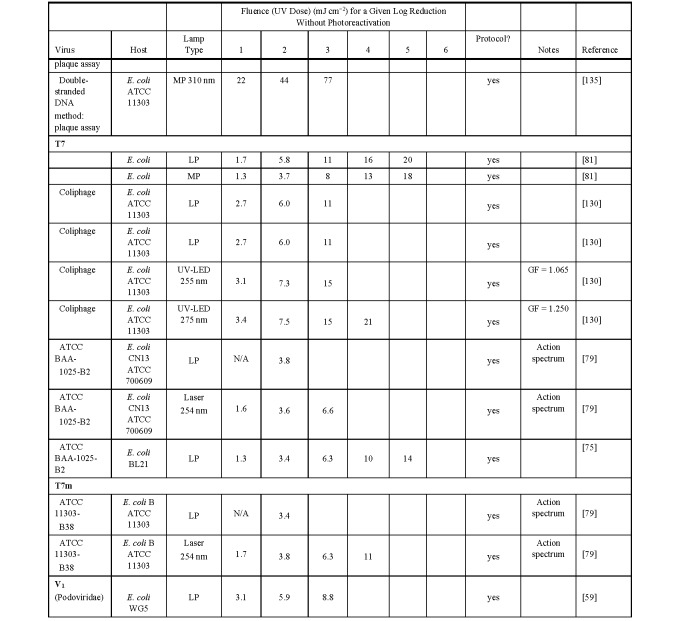

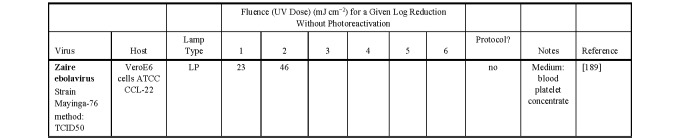
Fluences for multiple log reductions for various viruses

a Median Tissue Culture Infectious Dose

b DNA weighted fluence

c Long-Range Quantitative Polymerase Chain Reaction using a 6 kilobase fragment of DNA

d Long-Range PCR using a 1.1 kilobase pairs fragment of DNA

e Action spectrum weighted fluence

f Quantitative PCR

g Integrated Cell Culture Quantitative PCR

h The water depth was only 2 mm, so the water factor would have been very close to 1.0. Thus, although the protocol corrections were not made, the corrections would have been small

i Integrated Cell Culture and Reverse Transcription PCR

j Madin-Darby Canine Kidney cells

k Multiplicity of Infection

l Buffalo Green Monkey Kidney cells

**Table A5:**
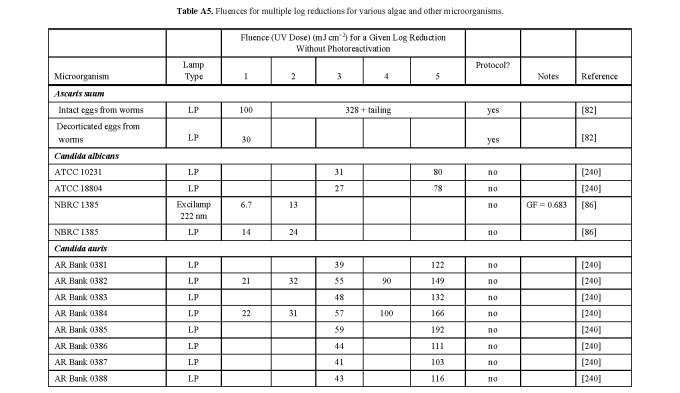

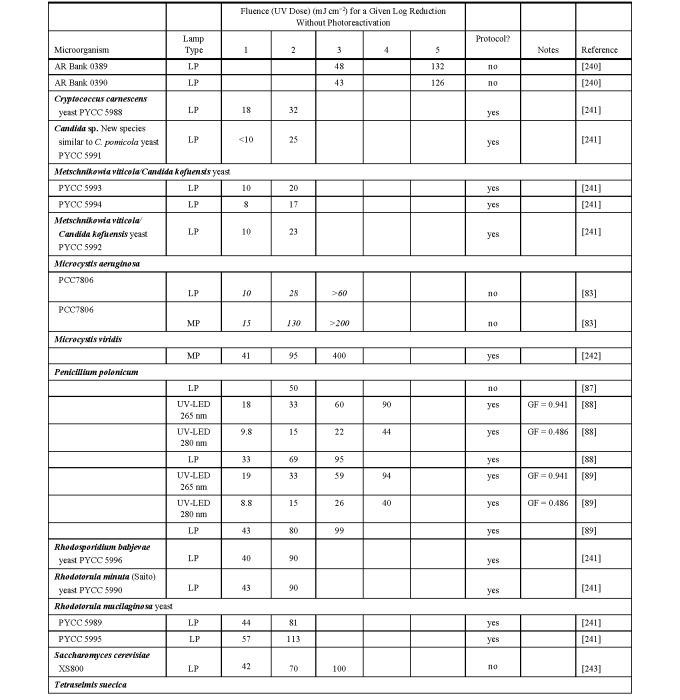

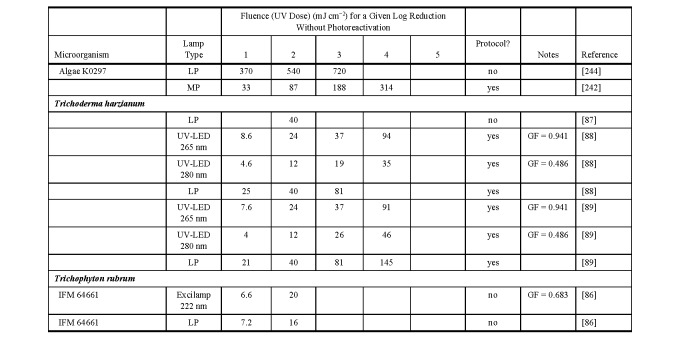
Fluences for multiple log reductions for various algae and other microorganisms.
